# Effect of Sorghum on Rheology and Final Quality of Western Style Breads: A Literature Review

**DOI:** 10.3390/foods10061392

**Published:** 2021-06-16

**Authors:** Rubina Rumler, Regine Schönlechner

**Affiliations:** Department of Food Science and Technology, University of Natural Resources and Life Sciences, Muthgasse 18, 1190 Vienna, Austria; rubina.rumler@boku.ac.at

**Keywords:** sorghum, western bread, rheology, baking, wheat sorghum blends

## Abstract

Due to the extreme weather conditions, caused by the climate change, the usual wheat harvest yield and quality in the Western countries were difficult to maintain in the past few years. The altered wheat quality was primarily characterized by a rising protein content. The application of high protein wheat flours in baking products leads to baking difficulties due to its elastic dough behavior. As these issues will further face the Western cereal cultivation, heat resistant cereals, like sorghum, are attracting increasing interest. A partial substitution of wheat with sorghum might offer one possible solution to address the current challenging situation. To introduce sorghum in the Western cereal and baking industry, the grain and its unique chemical and rheological properties need to be more widely promoted. Until now, several authors have conducted studies in order to emphasize the high potential of sorghum. The aim of the present review is to broaden the current knowledge of the chemical, rheological and baking properties of sorghum in comparison to wheat. The review further demonstrates promising approaches, which might be from interest in order to achieve higher wheat-sorghum bakery end product qualities.

## 1. Introduction

The wheat grain industry already experienced the effects of the climate change in the past few years. Dry climate with low rainfall was responsible for crop failures of wheat. According to a study, 60% of the current wheat growing areas could be affected by insufficient water supply by the end of this century [1]. However, due to the climate change not only wheat harvest losses happened, but also the wheat protein contents increased in the last few years [2]. A suitable solution must be found to compensate for the loss of wheat in the grain and baking industry and to deal with the altered quality. A possible way to address these challenges could be the use of food additives or to import wheat from other countries. A more sustainable, customer-friendly and innovative option could be the use of regional grains, preferable gluten-free grains, in wheat products. A partial replacement by gluten-free cereals might offer an option in order to compensate for the changed protein content of wheat. Sorghum, which is gluten-free and heat resistant, presents a promising grain in this context. The use of sorghum in wheat products could be a possible future solution to counteract the high climate-induced protein content of wheat. In addition, end products consisting of sorghum can gain valuable nutrients [3].

In order to introduce sorghum into the Western diet, the nutritional and technological challenges have to be considered. Mainly the altered protein composition, due to the absence of gluten results in changed dough rheological parameters and end-product qualities [4,5,6,7]. However, like other cereals sorghum contains tannins, trypsin inhibitors and phytic acids, which are known to impair protein digestion and mineral absorption [8]. Sometimes these values are higher than in other cereals. In addition, sorghum prolamins, called kafirins, are known to have a low digestibility, especially in prepared foods [9], which is mainly due to their crosslinking properties upon processing. Sorghum possesses higher crosslinking properties than wheat for example. However, if this protein cross-linking could in turn offer beneficial viscoelastic properties in food is still unknown [10].

This review summarizes the main chemical properties of sorghum and highlights its unique nutritional profile, reveals the current state of research on the use of sorghum in wheat doughs and suggests promising technologies that could lead to improvements in wheat-sorghum doughs.

## 2. Classification and Morphology of Sorghum

**Figure 1:**
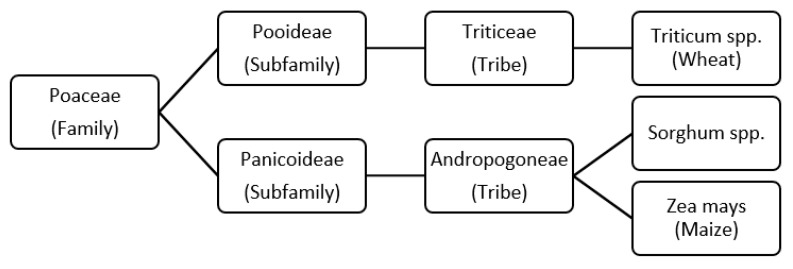
Family tree of sorghum. Modified according to Taylor [12].

Although sorghum is not well known in the West, it is the fifth most produced cereal worldwide. In Africa sorghum is considered to be the most important cereal. Outside of Africa production takes place mainly in India, USA, Australia, and Argentina, whereby sorghum in the USA and in Australia is mostly used as animal feed. Five races are known under sorghum: *bicolor*, *kafir*, *caudatum*, *durra* and *guinea*. All combinations from those mentioned races are called intermediate races. Of all races, *Sorghum bicolor* Moench is mainly cultivated [11]. According to Taylor there are different classification systems of sorghum. Some of them seem to be complex. However, like wheat and maize sorghum is classified into the grass family *Poaceae* as can be seen in Figure 1. Sorghum belongs, like maize, to the subfamily *Panicoideae* and to the tribe *Andropogoneae* [12]. Due to the botanical relationship sorghum is often compared to maize.

**Figure 2:**
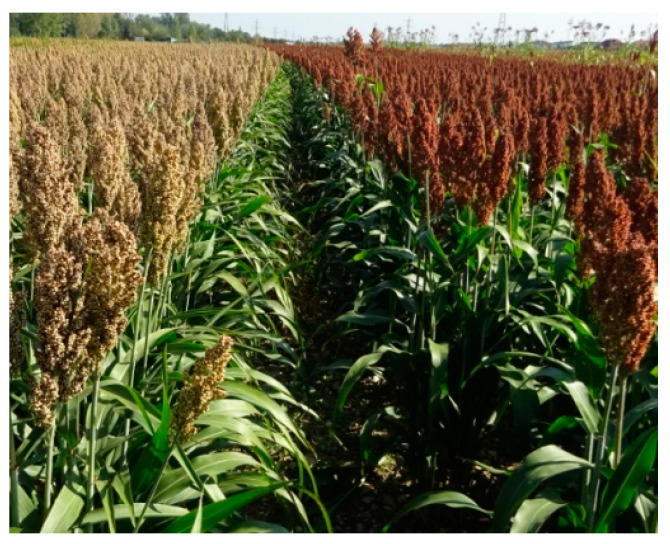
Sorghum field in Hörsching, Austria (©Rumler, 2020).

The structure of a sorghum kernel is comparable to the structure of a corn kernel, but in general, a sorghum grain is smaller. Awika et al. [13] determined the diameters of different sorghum kernels to range between 1.7–2.7 mm, and the thousand kernel weight of different varieties was between 15.2–38.8 g. The extent of the pericarp thickness and the presence of a testa is variety dependent [13]. Both sorghum and maize, have an endosperm with a floury and a corneous part [14]. Sorghum kernels appear in color nuances of white, red, brown and black (Figure 2).

However, the color of the pericarp is not always indicative of the appearance of sorghum flour. For example, a sorghum kernel which owns a red pericarp, may have a light endosperm color. Therefore, if special food colors are required, the insertion of sorghum fraction flours may be useful in order to achieve appropriate food appearances [15].

## 3. Chemical and Nutritional Properties of Sorghum

### 3.1. Protein

The protein content in sorghum can be comparable to the protein content of wheat [5,16]. However, Tasie and Gebreyes [17] found a wide range of 8.20–16.48% between different sorghum whole grain varieties. Attempts have often been made to identify correlations between nutrients and the sorghum kernels color. Whether the protein content differs between red and white sorghum is still poorly understood. A research of Galan et al. [18] detected a higher protein content in red whole grain sorghum flour, while Yousif et al. [19] and Srichuwong et al. [20] figured out a higher protein content in whole grain white sorghum flour.

In sorghum protein the predominant fraction is the prolamin fraction, which is called kafirin. According to a study almost half of the sorghum protein belonged to the kafirin fraction (47.7%), followed by the glutelin fraction (24.1%) and the albumin and globulin fraction (18.8%) [21]. Regarding the kafirins, four types of kafirins, occurring in form of protein bodies, can be mentioned: alpha-, beta-, gamma-, and delta-kafirin. Alpha-kafirins are the most common kafirins found in sorghum [10,22]. Detailed amino acid analyses have been carried out by Mohapatra et al. [23]. The most common amino acids found in whole grain sorghum were glutamic acid (44.38 mg/100 g protein), arginine (28.34 mg/100 g protein), aspartic acid (20.50 mg/100 g protein), leucine (16.50 mg/100 g protein) and glycine (16.22 mg/100 g protein) [23]. The limiting acid is lysine, as for most other true cereals [24].

Sorghum proteins have been gaining much attention due to their ability to crosslink, in particular upon cooking. Authors believe that this kafirin cross linking is connected to low protein digestibility of sorghum. In this context a comparison of the digestion of kafirins and zeins was performed by Emmambux and Taylor [9]. The authors analyzed the digestibillity of sorghum and maize meal as well as the digestibillity of aqueous tertiary butanol extracted kafirin and zein. In order to evaluate the influence of heat on sorghum and maize digestibillity, the samples were undertaken a boiling and a pressure cooking treatment. Already in the untreated state, the maize meal and extracted zeins showed a higher digestion rate than the sorghum meal and kafirins. However, after boiling and pressure cooking this difference was even more obvious. Authors detected that sorghum kafirins (obtained from decorticated sorghum kernels) were more polymerized by disulfide bridges during the cooking process than the zeins [9]. Same authors further pointed out, that sorghum proteins were not only able to form complexes between each other, but also had the ability to connect with condensed tannins and tannic acids [25]. This leads to the suggestion that sorghum kernel dehulling or the usage of condensed tannin free sorghum varieties might offer a higher digestibillity. However, crosslinking interactions According to Hamaker and Bugusu [10] sorghum proteins are embedded within protein bodies and therefore hard to access, but on the other hand they may show viscoelastic behavior in food. However, several questions remain unanswered at present. Fundamental investigations are still needed in order to determine the properties of kafirins.

### 3.2. Carbohydrate

Carbohydrate amounts between 67.5–76.4% were found in different whole grain sorghum varieties from Ethiopia [17]. Pontieri et al. [26] found that five varieties of refined sorghum, which were grown in Italy, the USA or Uganda, had an average carbohydrate content of 75.23% in dry matter. Starch is the main component of carbohydrates and is located in the endosperm. Srichuwong et al. [20] reported a starch content of 72.2% and 73.8% in dry matter in white and red whole grain sorghum, with amylose contents of 25.8% and 24.6% respectively. However, there is a wide range of amylose content in sorghum varieties, and even waxy varieties, which have high amylopectin contents occur in the wild [27]. The diameter of starch granules was measured to be between 5–35 µm and they appeared as polygonal and spherical [20], but also more unusual granules shapes, which researchers called “doughnut-shaped”, were occasionally discovered in sorghum kernels [28]. Miafo et al. [29] determined the content of free sugars in sorghum refined flour of 30.35 mg/g sorghum flour. Sucrose made up the largest proportion (12.42 mg/g), followed by maltose (4.25 mg/g) and fructose (4.22 mg/g). The usage of sorghum flour might offer a sugar reduction possibility in bakery products as it has already a unique sweet taste itself. As humans are tending more and more towards balanced diets, sensory trials are required in order to state if sorghum can partially replace household sugar.

Whole sorghum contains a notable amount of dietary fibers, which is mostly located in the outer layers of the sorghum kernel. A study carried out in 2012 showed a total dietary fiber content between 9.13–15.09 g/100 g that has been detected in eight sorghum varieties, whereby the soluble fiber part ranged between 0.15–0.88 g/100 g [30]. Authors compared the fiber content determined in whole grain sorghum, dehulled sorghum and sorghum bran. It was found that the bran fraction had the highest value with 41.38 g/100 g, followed by 26.34 g/100 g in whole grain sorghum and 11.53 g/100 g in hulled sorghum [31].

### 3.3. Fat

Whole grain sorghum flour is known for its high fat content (3.5%) especially compared to wheat (2% in whole wheat) [3]. Fat is mostly located in the sorghum germ (Taylor, 2003). The most common fatty acids are linoleic acid (18:2), followed by oleic acid (18:1) and palmitic acid (16:0), with average contents of 43.86%, 37.98% and 13.07% in refined sorghum flour, respectively [26]. The ratio of saturated to unsaturated fatty acids was found to be 0.27–0.37 [32]. Knowledge is lacking if high fat contents in sorghum flour play a crucial role within shelf life. However, [33] were able to extend the storage time of sorghum flour from 15 days to several months by means of heat treatments. As sorghum is mostly harvested in Africa, knowledge about sorghum milling in the Western countries was rare until very recently. It was not clear whether a high fat content in sorghum brings difficulties within a milling process. However, in 2021 authors concluded that sorghum flour can be provided via commercial wheat mills [15].

### 3.4. Micronutrients

Sorghum is known for having a valuable micronutrient profile. Istianah et al. [5] determined 1.64% ash in dehusked sorghum. However, it seems that micronutrient contents vary to a great extend among different varieties [17,34]. Tasie and Gebreyes (2020) identified ash contents between 1.1–2.3% in dry matter of different whole grain sorghum flours from Ethiopia. Regarding the micronutrients, amounts of phosphorus ranged between 112.5–327.7 mg/100 g, of sodium between 2.2–6.2 mg/100 g, of magnesium between 62.0–207.5 mg/100 g and of calcium between 9.5–67.2 mg/100 g [17]. Varieties with a particularly high mineral content exist, like for example the variety Tabat with phosphorus quantities of 350 mg/100 g, sodium of 14.5 mg/100 g, magnesium of 329 mg/100 g and calcium of 24.5 mg/100 g extracted sorghum flour [35].

### 3.5. Secondary Plant Products

Although people in the Western hemisphere are increasingly turning to alternative cereals with valuable ingredients, like amaranth or buckwheat, sorghum is still unknown. However, sorghum also offers a lot of promising health-promoting components. A review pointed out that especially sorghum polyphenols, as they have antioxidative effects, can help preventing chronic diseases, like improvement of insulin sensitivity, reduction of fat accumulation, or reduction of mild chronic inflammation [36]. Therefore, many research studies are interested in measuring the polyphenol content of sorghum, regardless of the research question. It can be stated with certainty, that polyphenols vary enormously among the biodiversity of sorghum. However, despite this interest, little research tended to focus on the context between polyphenols and sorghum colors. While researchers found that the polyphenol content of whole grain wheat (92.4 mg/100 dry matter) and whole grain white sorghum (95.2 mg/g dry matter) was similar, whole grain red sorghum had a significantly higher content of 203.5 mg/g dry matter [20]. Higher polyphenol levels in whole grain red sorghum flour than in whole grain white sorghum flour were confirmed by further studies [19,37]. If polyphenols are determined in more detail, flavonoids and phenolic acids are mainly mentioned. Wide ranges of phenolic acids were reviewed in the past. However, in red sorghum, as well as in white sorghum, ferulic acid was found to be the most predominant phenolic acid [38]. Among all flavonoids, special attention has been paid to the tannins, chemically named proanthocyanidins. Tannins are known to reduce sorghum digestibility [8]. There are low tannin and high tannin sorghum varieties, both naturally occurring. Tasie and Gebreyes [17] examined 35 whole grain sorghum flours from Ethiopia and detected tannin contents between 1.3–3337.2 mg/100 g dry matter. They also reported that condensed tannins and tannin acids in sorghum can form complexes with kafirins. Such a complex formation is one reason to lower sorghum protein digestibility. There was no evidence that other phenolic compounds, such as catechin or flavonoids, can bind kafirin [9]. A decreased protein digestion of tannin containing sorghum compared to non-tannin sorghum was also detected by Wedad et al. [39]. As condensed tannins are not present in wheat [14], it is questionable if using tannin rich sorghum varieties in a wheat blend leads to nutritional advantages. Besides phenolic acids and flavonoids, carotenoids also offer antioxidant properties. Luteolin, zeaxanthin and beta-carotene were found in amounts of 122.3 mg/kg, 25.2 mg/kg and 27.0 mg/kg, respectively, in white sorghum [40].

## 4. Physical Properties and Dough Rheology of Wheat-Sorghum Blends

It is known in general that addition of non-gluten containing flours to wheat doughs changes the final dough properties mainly due to the dilution of gluten, or the reduction of the final gluten content, respectively. However, also other grain components, e.g., the starch content, the content of plant secondary metabolites or the “new” protein composition, have important effects on processing. Based on these premises the current knowledge of the effects of sorghum addition on the physical properties and dough rheology of sorghum-wheat blends shall be summarized, as far as it has been investigated so far. Only a few studies were performed in this respect.

Knowledge of dough rheology allows to estimate final product quality. The instruments to investigate dough rheological behavior are the farinograph, mixograph (information on dough preparation, mixing and handling properties), and extensograph or alveograph for measurement of the rheological properties of the dough after mixing (information about elasticity). These instruments measure power input during dough preparation caused by either mixing action or extensional deformation. Originally these devices have been developed to determine wheat flour. Although it allows to investigate blends of wheat with non-wheat flours, it has its limitations at higher amounts of addition.

### 4.1. Thermal Properties of Sorghum

Information on the thermal properties delivers insights into the principal behavior of flour upon processing and are mainly determined by Rapid Viscograph Analyser (RVA) or Brabender Amylographs©. Usually, these types of equipment describe the thermal properties of starch suspensions, which is highly influenced by the starch composition like amylose and amylopectin ratio, but also by other grain components as it is recognized more recently. While both devices measure the pasting viscosity, only the RVA includes a cooling phase. Through this cooling phase information about starch retrogradation can be observed through the final viscosity value. Further, the heating rate of both devices is different. The RVA is able to heat up faster, as it requires less sample amount compared to the Brabender Amylograph©.

Ragaee and Abdel-Aal [41] examined the starch pasting properties of soft wheat and whole grain sorghum using an RVA. In general, the peak viscosity and final viscosity were higher for soft wheat than for sorghum. However, with 94.9 °C the pasting temperature for sorghum was similar to the pasting temperature of wheat. Hugo et al. [42] confirmed that wheat flour had higher RVA values than sorghum flour, but interestingly showed that sourdough fermentation and re-drying of sorghum flour increased the RVA data towards those of wheat flour. Preconditioning of sorghum might thus be necessary to achieve optimized rheological parameters. These data suggest that blending wheat flour with sorghum flour will result in a decrease of the resulting properties, but some research studies found opposite results. Ragaee and Abdel-Aal [41] added 15% sorghum to wheat flour and found a much higher final viscosity for the blend than for 100% soft wheat [41]. Such high final viscosities of wheat-sorghum blends underline high end product firmness of wheat-sorghum breads [7]. In addition to RVA measurements, a few researchers used an Amylograph to determine pasting or gelatinization properties. An Amylograph usually measures the suspension in a temperature range from 30 to 95 °C only, and thus shows begin of gelatinization (°C), peak viscosity and gelatinization or pasting temperature (°C). Sorghum flour showed a pasting temperature of 79 °C and a peak viscosity of 310 B.U [43]. Blending wheat flour with sorghum flour decreased peak viscosity with increasing sorghum addition, also measured using a Brabender Amylogram^©^ [3]. While 100% wheat flour gave a peak viscosity of 585 B.U., addition of 15% whole grain sorghum flour reduced it to 500 B.U., 30% whole grain sorghum flour to 430 B.U. Istianah et al. [5] pointed out after their study that low amylose levels are associated with low final viscosity. A discussion on whether waxy, hetero-waxy and non-waxy sorghum varieties have different thermal pasting properties already existed in 1988. Authors assumed that at least two waxy alleles must be present to significantly change the thermal starch properties [44]. Sorghum can greatly differ in amylose-amylopectin ratio and alpha-amylase activity [45]. As alpha amylase is contributing to desired bread quality [46], it might be interesting to investigate the connection of alpha amylase activity to sorghum bread quality. Overall, until now, there has been no detailed investigation about how sorghum starch properties influence baking products.

Srichuwong et al. [20] found evidence of the influence of non-starch components on starch gelatinization. When comparing the thermal starch properties of whole grain sorghum flour and isolated sorghum starch, it was found that the pasting properties of whole grain sorghum flour were lower than those of isolated starch. These authors pointed out that proteins might play a role, and other studies have further shown that phenolic compounds [37,47,48] and endosperm texture character [48] influenced the starch pasting properties of sorghum.

### 4.2. Water Absorption and Mechanical Stress Measurement of Wheat Sorghum Doughs

Attempts have been made already to understand how sorghum behaves in (leavened) dough. As it can be seen in Table 1 several studies have measured the water absorption and mechanical stress of wheat-sorghum blends using a Brabender Farinograph©. They all have shown that with increasing sorghum addition to wheat, the farinograph water absorption was decreased [3,6,7,16,19,49]. Only one study found opposite results, but did not indicate whether the results were significant [4]. Researchers showed that water absorption of sorghum-wheat flour blends were depending on the degree of flour fineness and starch damage [6]. However, summarizing the water absorption results, it can be assumed that sorghum needs less water than wheat for dough development. A limiting factor about this observation is that reliable data from the Brabender Farinograph© can only be obtained for wheat. There is little agreement on wheat-sorghum dough development. While two studies concluded that the dough development time decreased [49,50] the more sorghum was added, other researchers found that dough development time increased with increasing sorghum addition to wheat [6,7]. Therefore, there is no solid evidence whether sorghum requires a longer kneading time for dough structure development. Yousif et al. [19] pointed out that the rheological properties were dependent on the sorghum variety, as the dough development time of 30% whole grain sorghum flour in wheat blends was much longer for a white sorghum variety than for a red sorghum variety. This research was the only one, which carried out farinograph tests with doughs from blends of 50% wheat and 50% sorghum. When half of the wheat was substituted with white sorghum, dough development time decreased to the half compared to the control dough. A 50% substitution with red sorghum induced a slight decrease regarding the dough development time. More consistent results within the farinograph studies could be seen with regard to dough stability. The more sorghum was added to wheat flour, the shorter was the stability of the doughs [5,7,19,49,50]. This leads to the assumption that sorghum addition to wheat dough is less stable against kneading than a 100% wheat flour dough. Thus, sorghum might require shorter and/or less intensive kneading compared to wheat. However, sometimes the trend from lowest to highest sorghum addition showed an irregular trend [3,16], and according to Ognean [6], 40% coarse-grained sorghum flour in wheat flour showed better dough stability than the control dough (100% wheat). In general, the farinograph data were improved by coarse-grained sorghum flour compared to fine sorghum flour [6]. Dough softening with an increase in the percentage of sorghum was higher [3,7,49], although in two publications specific additions of 5% [4] and 15% [16] to wheat were able to improve dough softening. A higher dough softening with sorghum addition underlines the results of the dough stability: doughs consisting out of wheat and sorghum seem to be less resistant against kneading force. To conclude, these farinograph data of sorghum-wheat blends (see Table 1); sorghum flour addition to wheat flour decreased water absorption and dough stability, but no clear trend for dough development time or dough softening was observed, although the latter was sometimes increased. These inconsistent results can most likely be related to the fact that different flours and varieties have been used in these studies, which is true for wheat flour (different flour types and varieties) as well as for sorghum. In addition, particle size and starch damage of these flours might have varied [3,4,6,7,19,50]. Another important fact to consider is that this device was developed for the characterization of (the baking quality of) wheat flour. It is questionable whether the results of the Farinograph© test are suitable for determining the processing quality of sorghum, as they are for wheat. Torbica et al. [51] investigated for the first time whether farinograph data of wheat-sorghum blends can be transferred to end product quality of wheat-sorghum bread. Correlations could be identified mainly for 10% sorghum in wheat, where dough development time and dough stability correlated with specific volume and bread firmness, while water absorption and dough softening showed no correlation. Dough softening mostly correlated with 30% wheat-sorghum breads, prepared with a low kneading intensity. The authors stated that using a Mixo-doughLAB system seemed to be more suitable to predict a wheat sorghum bread quality [51].

Determining the water absorption behavior is principally also possible without any device. For this purpose, often the water absorption index (WAI) is mentioned. Al-Rabadi et al. (2012) measured the WAI with the same temperature as for the Farinograph test (30 °C). The WAI was 2.56 g/g for sorghum flour with a particle size of <125 µm. The effect of changed particle size could not be clearly defined [52]. Dayakar Rao et al. [53] investigated the WAI of different sorghum flour particle sizes at a temperature of 90 °C. Here it was shown that the WAI of sorghum flour milled with a hammer mill increased with decreasing particle size. Dayakar Rao et al. [53] stated that the WAI is depending on the milling method. Adebowale et al. [54]. measured the WAI (at 30 °C) of sorghum-wheat blends containing 5%, 10%, 15% or 20% sorghum. No linear trend could be observed the more sorghum was added to wheat, but the 20% sorghum-wheat blends had the lowest WAI values. Overall, these results point out that determination of a reliable WAI-value of sorghum flour might require different water temperatures than wheat. Future studies on the current topic are therefore needed in order to verify the best methodology for determining the WAI of sorghum.

### 4.3. Extensibility Properties of Wheat Sorghum Doughs

While at least a few studies about the mixing properties of wheat-sorghum blends were available, dough extensibility of sorghum blends was hardly investigated so far, which makes it difficult to derive general statements or directions for subsequent baking (Table 1). As sorghum contains no gluten like wheat, a decrease of extensibility after sorghum addition can be supposed. In the studies of Rizk et al. [4] and Seelem and Omran [16], who both applied a Brabender Extensograph© this expectation was confirmed, but only after a sorghum addition of 15% and more. With lower additions of 5% or 10% the dough extensibility was not changed compared to 100% wheat ([4] for 10%) or even increased ([16] for 5% and 10%). Dube et al. [49] found already a lower extensibility with an addition of 10% sorghum to wheat. Research is still needed in order to evaluate if interactions between wheat and sorghum compounds take place already if only small amounts of sorghum are added to wheat. However, all mentioned studies detected that dough resistance to extension fell linearly with increasing sorghum addition to wheat, already at low amounts. Abdelghafor et al. [50] recognized no consistent trend for dough extensibility as well as for dough resistance to extension. In consequence, the R/E-ratio showed differing results. While it fell within the blends with increasing sorghum content in the study of Rizk et al. [4], it increased in the study of Seleem and Omran [16] and it delivered an inconsistent trend again in the study of Abdelghafor et al. [50]. Regarding the energy required to disrupt the dough, all studies found a reduction with increasing addition of sorghum to wheat [4,16,49,50]. Transferring these Exentsograph© observations to practice, it can be assumed that sorghum addition to wheat may need less force in order to process the dough (e.g., pizza dough rolling or croissant molding). However, the fact that sorghum constituents may interact with wheat constituents (e.g., proteins) during fermentation, leads to inconsistent results. As mentioned before, the above cited studies were applied by a Brabender Extensograph©. There are also studies that used an alveograph, where the visco-elastic properties are determined by blowing the dough into a bubble and thus the dough’s extensibility, resistance to extension and the energy required to pop the bubble. In the study of Sibanda et al. [7], the extensibility (mm) and energy (cm^3^) of the dough decreased with increasing addition of sorghum to wheat, dough resistance (mm H_2_O), as well as R/E-ratio increased linearly with the addition of sorghum.

## 5. Effects of Sorghum Addition in Baking Products

### 5.1. Bread

As traditional sorghum products (e.g., beverages, porridges, flat breads) do not need network-forming proteins, not many studies about baking with sorghum, in particular leavened bakery products were undertaken in the past years. Reasons for using sorghum in wheat bakery products were, for example, to reduce imports of wheat in Africa [7,16].

As described in Chapter 4, some researchers determined rheological dough parameters for sorghum wheat blends. Consequently, some of these researchers analyzed standard bread properties consisting of wheat-sorghum doughs up to a maximum of 40% wheat substitute with sorghum [6,7,55]. The attempt of this chapter is to summarize the effects of sorghum on final bread quality produced from blends of wheat and sorghum, excluding the production of gluten-free breads (100% sorghum or in combination with other gluten-free flours). In Table 2, the most important effects were outlined. As can be observed, most researchers described the decrease of (specific) bread volume when wheat flour was replaced by sorghum flour, which seemed to decrease gradually with increasing sorghum amount. Significant reduction of loaf volume was detected after 15% sorghum addition by Elkhalifa and El-Tinay et al. [55], and by Sibanda et al. [7], who applied the straight dough method for breadmaking, after 20% sorghum addition. Higher ratios of sorghum in wheat blends (30% and 40%) were investigated by Sibanda et al. [7], Ognean et al. [6] and Angioloni and Collar [56]. Here, not only a bread loaf reduction was observed, but also diminishing effects on crumb porosity and elasticity. As crumb porosity is related to specific volume, the lowest specific volume and smallest crumb pore sizes were found in wheat bread with 40% sorghum. Further effects of sorghum addition described, were bread weight increase, darker crust and crumb color, as well as an increase in crumb firmness (see Table 2). Ognean et al. [6] found that sorghum flours particular size seemed to be important for final wheat-sorghum bread quality. Fine sorghum flour had a greater porosity reduction effect, than coarse sorghum flour with a larger particle size. In addition, crumb elasticity of wheat sorghum breads with coarser sorghum flour was less affected as with fine sorghum flour.

Apart from the physical properties of wheat-sorghum bakery products, also sensory trials were undertaken, which appeared to be promising. Breads with up to 30% sorghum were evaluated to be similar to the control breads (100% wheat) in terms of aroma, texture and taste in the study of Sibanda et al. [7]. In addition, flat breads with up to 30% sorghum were rated equally to flat breads from 100% wheat [16]. However, it has to be considered, that these sensory tests were carried out in Zimbabwe and Egypt, where people are familiar to the taste of sorghum. It is not known yet how bakery sorghum containing products will be perceived by consumers to whom sorghum is new. This task might be challenging for future integration of sorghum into Western food-based markets.

Overall, it can be concluded that a small amount of sorghum can be used in wheat standard products without any or only small loss of product quality and seems to be promising in terms of sensory properties. However, not many efforts have been made to optimize wheat-sorghum products. Adapting technological parameters, like water addition, kneading time or fermentation temperature, etc. was not investigated. Optimizing these parameters and further handling of wheat sorghum doughs will have major effects, and might lead to improved and acceptable final bread qualities. In addition, and as already mentioned, sorghum proteins might have viscoelastic properties in food, although probably not in their native form [10]. Studies to evaluate or modify these cross-linking properties of sorghum in bakery products are still missing. This task will require thorough and fundamental investigation.

### 5.2. Cakes

While at least a few studies have been published on wheat sorghum breads, research on using sorghum for other bakery products like cakes, pastries, or biscuits is even more scarce (see Table 2 for outline of findings). Sponge cake and biscuits including sorghum were investigated by Rizk et al. [4]. An addition of up to 15% sorghum in wheat sponge cake (made from flour, egg, sucrose, baking powder, vanilla and water), induced no change in weight and volume of the products. In sorghum blended wheat biscuits (made from flour, butter, sucrose, baking powder, vanilla and water) a weight change was observed neither with increasing sorghum content, but volume was negatively affected with gradual addition. The overall sensory acceptance was reduced for wheat-sorghum biscuits compared to the 100% wheat products, but still rated to be good [4]. As mentioned in the context of wheat-sorghum breads, the used flour might be critical to the properties of wheat-sorghum pastry products, as well. The study of Dayakar Rao et al. [53] indicated that biscuits baked from 100% coarse sorghum flour (particle size 251 µm) showed significant higher thickness, softer texture and lighter color properties than biscuits made from 100% fine sorghum flour (particle size 75 µm).

For pastry and other fine bakery goods it has to be taken into account that they contain a wider and very heterogenous range of ingredients compared to bread, thus, the effect of sorghum addition will cause rather varying effects on end-product properties. On the other hand, this larger number of ingredients can also act positively, as the quality of fine bakery products is less determined by the flour component than in bread. This seems to facilitate the incorporation of sorghum into wheat cakes/biscuits/pastries. Overall, the introduction of sorghum into this product category could be a useful strategy to slowly adapt the Western consumers to sorghum.

## 6. Introducing Sorghum into the Western Diet: Evaluation of Potential

As summarized in Section 5, knowledge and research on using sorghum in Western style (leavened or fine) bakery products is yet very scarce. Still some ideas or approaches can be derived.

- Sorghum variety selection

A first objective of baking with sorghum will be careful selection of sorghum variety, in particular with regard to polyphenol content (tannins). However, although a wide range of sorghum varieties were thoroughly analyzed for its chemical composition, information on using different sorghum varieties in rheological and baking trials is still only limited available. Interesting findings were revealed when tannin-containing and non-tannin-containing sorghum varieties were compared in baking trials, where fermented sorghum flat bread from non-tannin sorghum flour had improved end product properties over tannin-containing sorghum flour [57]). In another major study, Akin et al. [58] investigated the addition of zein to sorghum bread in order to obtain a higher bread quality. Here different sorghum varieties were used for the investigation. The highest bread quality was achieved when a white non-tannin sorghum flour was used. Breads consisting out of higher polyphenol sorghum flours showed lower bread quality [59]. Hence, finding suitable sorghum varieties which provide optimized baking properties might be necessary in order to facilitate the incorporation of sorghum in Western style bread.

Apart from this, influences of other ingredients like protein, starch and dietary fiber composition and properties, are still much under-researched. As the amylopectin and amylose ratio in sorghum can vary to a great extent, attempts are still needed in order to evaluate suitable starch properties for sorghum baking [45].

- Sorghum milling and fractionation

Besides raw material properties, appropriate grain processing methods have to be chosen and adapted to the use of sorghum. Production of (whole meal) flour or flour fractions and therefore implementation of milling systems plays a crucial role on end-product quality. The improvement of bread loaf volume [6], or the height, texture and color of biscuits [53], when using courser sorghum flour with larger particle size, compared to finer flour was described in the sections before. Different effects seemed to be achieved by application of different milling systems, as Dayakar Rao et al. [53] found an Indian traditional grinding system to be superior to a hammer mill.

Evidence how different milling fractions act in wheat baked goods was found in gluten-free formulations. Trappey et al. [59] examined the effect of sorghum flour with different extraction rates (produced using a lab mill) on gluten-free sorghum bread volume. The lower the sorghum flour extraction rate, the higher was the bread volume and the lower the crumb firmness.

- Sorghum pretreatment

In African countries, where the use of sorghum stands a long tradition, specific processing methods have evolved, in particular for pre-preparation of sorghum, like soaking, fermentation (using a large range of starter cultures), sprouting, malting and thermal pre-treatment. This overview should highlight, that introducing new products into existing dietary patterns often requires new and innovative processing methods. To survey traditional and long existing approaches that have been applied in African countries for centuries will be helpful to get an idea of potential strategies.

Lactic acid fermentation, in the case of bread sourdough fermentation, is also known in the Western countries, moreover it was only recently gaining increased interest again. A few studies are available that undertook efforts to investigate the effects of sourdough fermentation for baking with sorghum. Hugo et al. [42] and Istianah et al. [5] have recognized that the use of fermented sorghum flour (fermented, dried and re-milled) improved wheat sorghum breads with a 30% sorghum content. While Istianah et al. (2018) fermented sorghum with dry yeast and *L. Plantarum* for 24 h, Hugo et al. (2003) prepared sour dough with a natural starter, which was back-slopped from previous trials. According to Karrar et al. [60] sorghum-wheat breads applying sorghum sourdough showed better (specific) volume than 100% wheat control doughs (without sourdough fermentation), even at high amounts of 30% sorghum sourdough. However, breads showed its highest (specific) volume with 20% sorghum sour dough. Regarding gluten-free breads, Wolter et al. [61] found a negative influence of sourdough fermentation. Although some research has been performed on sourdough fermentation of sorghum, it has still not been sufficiently investigated in detail for future application.

The principle of thermal pretreatment has been investigated as a rather nonconventional strategy for baking by Marston et al. [62], who applied the extrusion cooking technology for gluten free baking and found improved end-product qualities. Gluten free cakes and breads with extruded sorghum flour had a softer texture, higher volume and better consumer acceptance. Another study showed improved sensory properties after incorporation of high pressure treated sorghum flour (40%) into wheat bread compared to the incorporation of untreated flour (40%). However, no improvements on physical bread quality (texture or volume) were observed [56].

Aside from processing adaptation, for food development of sorghum, its chemical and nutritional composition should be considered. As described before, wheat and sorghum have comparable macronutrient compositions, which allows similar food uses. However, in terms of micronutrients, sorghum varieties differ substantially from wheat as they mostly contain higher amounts of minerals and secondary plant metabolites. For future food use, attention must be paid to the tannins and phytates, as without pre-treatment of sorghum they can negatively influence the nutrient profile of food products when high amounts of sorghum are added [25]. On the other hand, a partial replacement of wheat by sorghum allows a significant enrichment of beneficial nutrients, especially in food products from refined flours that otherwise were rather poor in these nutrients. A reduction of trypsin inhibitors and phytic acids can be obtained by selection of specific varieties and by employing some of the above mentioned procedures, like fermentation, germination and soaking. These are also capable to reduce the amount of tannins in sorghum as was found by Osman and Gassem [8] with the exception of germination. Fermentation reduced trypsin inhibitor activity, tannins and phytic acid of *Kisra* (a traditional African flat bread) [63]. and improved digestibility of sorghum [42]. Further researchers attempted to evaluate the impact of fermentation of sorghum on the nutritional profile. Here it was found out that as fermentation can decrease the phytate amount and phenolic compounds and provide a higher accessibility of iron the application of sorghum fermentation might be necessary for sorghum varieties rich in antinutrients [64]. A review by Pinelli et al. [65] summarized effects of different approaches in order to achieve higher sorghum bread quality (gluten free bread). Among other things it was pointed out that germinated flour may be helpful to achieve softer bread crumb, sourdough may improve crumb appearance and addition of native cassava starch addition may reduce crumb firmness.

## 7. Conclusions

Harvest data in the past years have shown that the quality of wheat has changed due to the effects of climate change. It can be expected that wheat yields will continue to decline in the coming years [2] and wheat quality to alter. In order to avoid wheat imports or the increased use of food additives, the diversity of raw materials should be extended. Within the cereal sector, sorghum shows great potential in terms of climate adaptability. The incorporation of sorghum within Western style products seems also promising for human nutrition, as preliminary research data suggest. Rheological studies performed with wheat sorghum blends showed that no consistent trend with gradual wheat replacement could be found, which might be an indication that strengthening effects between wheat and sorghum take place. With lower amounts of sorghum incorporation studies showed that acceptable, sometimes even improved, bakery products, were obtained [7]. However, thorough research is still necessary to determine optimum baking conditions, and even to define the milling procedure in order to produce sorghum flour properties that meet the demands for a wider use in wheat/sorghum blended bakery products. Application of sourdough technology seems to have a great potential [5,60], but also innovative and unconventional strategies need to be contemplated. Aside from the climate change implications, addition of sorghum allows to improve the nutritional profile of wheat products [3,16] Nevertheless, also in this respect further research is required in order to investigate and modulate (protein) digestibility of sorghum upon processing. For future integration of sorghum into the Western diet, a wide and unbiased approach covering all aspects of the food supply chain has to be pursued, including selection of sorghum varieties from all areas, fundamental chemical and nutritional investigations, extensive processing trials and open-minded consumers in order to achieve this ambitious aim.

## Figures and Tables

**Table 1:** Rheological characterization (Brabender Farinograph© and Brabender Extensograph©) of wheat-sorghum blends with increasing sorghum addition.

Parameter	Decreasing Value	Increasing Value	Irregular Trend
Water absorption (%)	X [3,6,7,19,49,50]	X [4]	
Dough development time (min)	X [49,50]	X [6,7]	X [3,4,19]
Dough stability (min)	X [4,7,19,50]		X [3,16]
Dough softening (B.U)		X [3,7,49,50]	X [4,16]
Dough extensibility (mm)	X [49]		X [4,16,50]
Dough resistance to extension (B.U)	X [4,16,49]		X [50]
Resistance/Extensibility Ratio	X [4]	X [50]	X [16]
Energy (cm^2^)	X [4,16,49,50]		

**Table 2:** Effects of sorghum incorporation into wheat products on selected quality parameters.

Product	Sorghum (%)	Findings How Sorghum Influenced Wheat Standard Products	Reference
Bread	15%	Volume and specific volume decreased, weight increased	[55]
Bread	30%	Volume decreased, crust and crumb color got darker, pores became irregular	[7]
Bread	30%	Volume decreased	[49]
Bread	40%	Volume decreased, porosity decreased, elasticity decreased	[6]
Bread	40%	Specific volume decreased, crumb firmness increased, cohesiveness decreased	[56]
Biscuit	15%	Sensory characteristics decreased	[55]
Biscuit	15%	Volume and specific volume decreased, width decreased, sensory characteristics decreased	[4]
Sponge cake	15%	Sensory characteristics decreased	[4]
Flat bread	15%	Freshness over storage decreased	[16]
